# Expression Activity of Artificial Promoters for Disease Resistance in Transgenic *Eucalyptus urophylla*

**DOI:** 10.3390/genes13101813

**Published:** 2022-10-07

**Authors:** Zhenchi Huang, Qingchun Xu, Xiaolan Fang, Zhihua Wu

**Affiliations:** 1 School of Life Science and Technology, Lingnan Normal University, Zhanjiang 524048, China; 2 Research Institute of Fast-Growing Trees, Chinese Academy of Forestry, Zhanjiang 524022, China

**Keywords:** eucalyptus, improved promoter, expression activity, position effect, tobacco mosaic virus, transgene

## Abstract

The transcriptional properties of artificial promoters are closely related to the type and arrangement position of cis-elements. GWSF (374-bp) was an effective SPIP with four cis-element dimers. There were four pathogen-inducible cis-elements in the GWSF promoter (GST1-boxes, W-boxes, S-boxes, and F-boxes) and a minimal cauliflower mosaic virus 35S promoter. V-element dimers were inserted into the upstream (VGWSF), midstream (GWVSF), and downstream (GWSFV) regions of the original GWSF promoter sequence to examine their affect on the position. The expression activity of promoters was analyzed and estimated using the histochemical staining of leaf discs of eucalyptus with transient expression, an image digitization method to extract the color features, and the induction treatment by a plant pathogenic microorganism/inducer and qPCR assays. The histochemical staining results of the adventitious buds indicated that the promoters had been successfully integrated into the *E. urophylla* genome and that they drove the expression of the *gus* gene. There was a noticeable difference in the intensity of color between the adventitious buds on the same callus block, as well as the intensity of color within the same adventitious bud. According to the established two-factor model of blue value, there was a greater difference between the levels of the genotype factor than the promoter factor in eucalyptus leaf discs. Further, the basal and inducible transcriptional levels of the three improved promoters were investigated by qPCR. With the basal transcriptional level of the GWSF promoter normalized to one, the relative basal levels of VGWSF, GWVSF, and GWSFV were 1.40, 1.45, and 4.15, respectively. The qPCR results were consistent with the staining results of GUS histochemical staining. The three improved promoters all had the properties of being induced by salicylic acid, *Ralstonia solanacearum*, and *Phytophthora capsici*. The three improved promoters demonstrated a significantly higher TMV induction activity: their induction activity from high to low was GWSFV > GWVSF > VGWSF. The findings will be beneficial to the construction and optimization of artificial promoters for transgenic plants.

## 1. Introduction

In tropical and subtropical regions, eucalyptus is the most widely grown hardwood crop due to its high economic benefits, high-stress resistance, and quick growth [1]. Eucalyptus is one of the three major species of trees used in plantations, along with pine and poplar. In light of the introduction of new varieties and the expansion of cultivation areas, it is imperative that their genetic quality and resistance be improved. Due to its high heterozygosity, long growth cycle, and incompatible barriers (self-incompatibilities), conventional hybrid breeding presents a challenge for eucalyptus [2]. In comparison with conventional breeding, transgenic breeding is more productive (cost-effective, time-efficient, and able to predict more desirable traits than conventional breeding). A successful transgenic breeding program begins with the screening of promoters with excellent transcription characteristics in eucalyptus cells to drive the expression of the target gene. As a woody plant, eucalyptus is difficult to obtain genetically modified plants that will be stable [3]. Therefore, the study of woody plant promoters was frequently conducted using model plants [4]. However, a promoter’s transcriptional characteristics are influenced by a number of factors, and the activity and inducibility of the same promoter in different species can vary significantly. Assays for transient expression mediated by *Agrobacterium* have the advantages of simplicity, rapidity, and a high transformation efficiency [5], which can be used to quickly and accurately determine the transcriptional characteristics of promoters in woody plant tissues [6].

Promoters are particular DNA sequences located upstream of the 5′ end of the coding regions, which initiate gene transcription by combining with RNA polymerase and regulate the expression mode and level of downstream structural genes [7]. Hence, the promoter region determines when and where the gene of interest will be expressed within the organism. Promoter activity and specificity are the two main parameters that regulate transgene expression. The selection of suitable promoters for the development of transgenic crops is one of the most important aspects of the process [8]. To regulate precisely the expression of resistance genes in plant disease resistance genetic engineering, ideal promoters with a low basal expression, wide induction factors, and high induction efficiency are required. As a result, ideal promoters minimize side effects and improve resistance to pathogens quickly [9]. Natural promoters often lack expression intensity or expression specificity, so it is difficult to achieve transgenic disease resistance by using them [10].

In plants, synthetic promoters introduced either stably or instantly are valuable for identifying functional regulatory elements and the transcription factors that govern the amplitude, spatial distribution, and temporal pattern of gene expression [11]. The sequence function of promoter elements has been studied in plants by fusing promoter fragments with reporter genes, such as CAT (chloramphenicol acetyltransferase) or GUS (glucuronidase) [12,13]. Identifying regulatory elements via promoter fragment analysis is still viable, but time-consuming and laborious. The use of synthetic promoters provides the possibility of designing expression requirements in accordance with the investigator’s requirements [14]. Functional verification of a putative cis-regulatory element can subsequently be carried out by constructing a synthetic promoter consisting of one to several copies of the element fused to a minimal promoter and reporter gene, followed by the introduction of the construct in planta [15,16].

**Figure 1:**
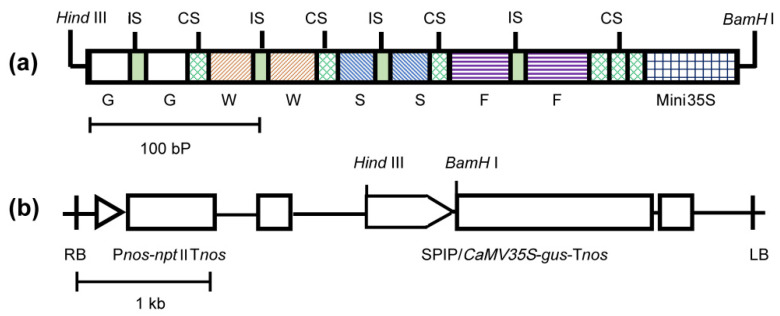
A diagrammatic sketch of GWSF in figure (**a**) and recombinant pB I121 harboring SPIP in (**b**) [7]. G: Gst1-box (5′-TTCTAGCCACCAGATTTGACCAAAC-3′) [22]; W: W-box (5′-TTATTCAG CCATCAAAAGTTGACCAATAAT-3′) [23]; S: S-box (5′-CAGCCACCAAAGAGGACCCA GAAT-3′) [24]; F: F-box (5′-TTGTCAATGTCATTAAATTCAAACA TTCAACGGTCAATT-3′) [25]; IS: 6 bp DNA insert sequence; CS: 10 bp DNA connection sequence; Mini35S: minimal *CaMV35S* promoter; P*nos*: nopaline synthase promoter; *npt* II: neomycin phosphotransferase II gene; *gus*: β-glucuronidase gene; and T*nos*: nopaline synthase terminator. (**a**,**b**) contain scales of 100 bp and 1 kb, respectively.

GWSF (374-bp), named for its four cis-element dimers, was a highly efficient SPIP (synthetic pathogen-inducible promoter) capable of causing rapid responses to a wide range of pathogens [7]. In the GWSF promoter, four pathogen-inducible cis-elements were present (GST1-box, W-box, S-box, and F-box) and a minimal cauliflower mosaic virus 35S promoter (−46 to +8 TATA box) (Figure 1). Our opinion is that plant pathogens primarily include bacteria, fungi, and viruses. The introduction of virus-inducible cis-elements in GWSF may make it possible for optimized promoters to exhibit virus-inducible characteristics. It has been shown that the 5′-flanking region of the tobacco N gene exhibits tobacco mosaic virus (TMV)-induced promoter activity and that the region between −290 and −271 contains a cis-element in response to TMV infection [17]. We referred to this region as the V-element.

Some studies on the activity of promoters revealed that promoters containing several types of cis-elements generally exhibit better expression characteristics than promoters containing only one type of element [18]. The strength of promoters can be increased by increasing the number of cis-elements [18], which is related to the increase in transcription factor binding sites [14]. However, spacing is crucial for synthetic promoter activity in at least one respect; when cis-elements were placed too closely together, their activity was lost [14]. Therefore, it is necessary to carry out experiments in order to determine the exact distance between the cis-elements. Considering that the same promoter element is relatively conserved across different plant species [19], combining dimers with different elements and the *CaMVmini35S* promoter [20] will lead to an ideal SPIP, allowing disease-resistant genes to be expressed precisely and in time [21].

This study aimed to evaluate the gene expression characteristics of different promoters and to find plant pathogen-inducible promoters with a low basal expression and high inducibility by salicylic acid, bacteria, fungi, and viruses. We designed VGWSF, GWVSF, and GWSFV promoters in this work, which are located in the upstream, midstream, and downstream regions of the GWSF promoter sequence inserted by a V-element dimer with virus-inducing activity [17]. An analysis of gene expression in transformed plants was carried out using the β-glucuronidase gene (GUS) as a gene fusion marker. The transcriptional levels of the three improved promoters in transformed adventitious buds were evaluated by the GUS histochemical assay. In eucalyptus leaf tissue, the transcriptional properties of the promoters were examined using a transient expression assay mediated by *Agrobacterium*. We also investigated the positional effect of newly added V-elements on the transcription properties of the original promoter. The results of this study will serve as a reference for the design of artificial promoters.

## 2. Materials and Methods

### 2.1. Plant Materials and Cultivation Conditions

Seeds of *E. urophylla* S.T. Blake were used for establishing the transformation method. Tissue-cultured seedlings of *E. urophylla* (EU6), *E. urophylla* × *E. grandis* (Guanglin 9), and *E. urophylla* × *E. tereticornis* (Guangzhou 1) were used for promoter selection, which were provided by the South China Experiment Nursery located in Suixi County, Guangdong Province. The one-year-old seedlings of three genotypes of eucalyptus were grown with vermiculite and cultivated in an artificial climate chamber at 28 ± 2 °C, 500 mmol/m^2^/s of light intensity, and photoperiods of 14 h light/10 h dark per day.

### 2.2. Improved Promoter Design and Vector Construction

The V-element sequence was 5′-TTGGGAAGGAATTTCCTACT-3′ [17], and a 6 bp ACTAGA sequence linked two V-elements to form a V-element dimer (Figure 1a). The V-element dimer was inserted into the upstream, midstream, or downstream regions of the GWSF promoter with a 10 bp GAAGATAATC interval sequence [7] to produce three new promoters, namely VGWSF, GWVSF, and GWSFV. The spatial distance between the V-element and the *CaMV35S* minimal promoter is 30 bp in GWSFV, 188 bp in GWVSF, and 330 bp in VGWSF, respectively. Two restriction sites (Figure 1a,b), *Hind* III and *BamH* I, were introduced into the upstream and downstream regions of the improved promoter sequence, which were synthesized by Shenggong Biotechnology Company (Shanghai, China) and cloned into the *pUC*19 plasmid. The improved promoters were used to replace the wild-type *CaMV35S* promoter in the plasmid pBI121 by restriction endonuclease digestion and ligase ligation in order to regulate the expression of the β-glucuronidase (*gus*) gene. As described in the literature [7], the construction of the plant expression vectors and the transformation of the *Agrobacterium* were conducted.

The success of the vector construction and transformed *Agrobacterium* GV3101 was identified by PCR allied with restriction endonuclease digestion and sequencing. The primers for PCR verification were as follows:

VGWSF forward/reverse: 5′-GCAAGCTTTTGGGAAGGAAT-3′/5′-TGGTGGCT GGATTATCTTCA-3′, the length of the amplified fragment was 215 bp; GWVSF forward/reverse: 5′-TGAAGATAATCCAGCCACCA-3′/5′-AGCGTGTCCTCTCCAAAT GA-3′, the length of the amplified fragment was 243 bp; GWSFV forward/reverse: 5′-CCAGAA TACTAGACAGCCACCA-3′/5′-GGAAGGGTCTTGCGGATTAT-3′, the length of the amplified fragment was 231 bp; and GUS forward/reverse: 5′-ACACCGATA CCATCAGG-3′/ 5′-TCACCGAAGTTCATGCCAGT-3′, the length of the amplified fragment was 480 bp.

### 2.3. Plant Tissue Culture and Genetic Transformation System

Viable seeds of *E. urophylla* were provided by the Research Institute of Fast-growing Trees, Chinese Academy of Forestry. After surface sterilization, seeds germinated on a half Murashige and Skoog (MS) agar medium for 8 days under darkness at 25 ± 1 °C.

The seedlings were illuminated at a photon flux density of approximately 20 μmol /m^2^/s for 1 d. Seedling hypocotyls were cut from the 9-day-old seedlings and inoculated on an SPCa medium [26] supplemented with 3.99 μmol/L of N-phenyl-N’-[6-(2-chlorobenzothiazol)-yl] urea (PBU), 0.57 μmol/L of 6-benzylaminopurine (BAP), and 0.57 μmol/L of indole acetic acid (IAA) (callus and bud inducible medium, CBIM), under darkness at 25 ± 1 °C. After pre-culturing for one week, the explants were immersed in the *A. tumefaciens* GV3101 solution for 30 min at −0.05 MPa, then were inoculated on the original medium to co-culture for 3 days in darkness. Subsequently, the explants were swirled in sterile water to remove *A. tumefaciens*. The explants were inoculated on the same medium with 200 mg/L of cefotaxime (Cef) for one week to decontaminate residual *A. tumefaciens*, followed by the CBIM with 200 mg/L of Cef and 50 mg/L of Kanamycin (Kan). The explants were sub-cultured every 2 weeks on a fresh CBIM medium until the adventitious bud regenerated from the callus, using 50 mg/L of Kanamycin and cefotaxime, gradually decreasing to 100 mg/L, under a 16 h photoperiod with a light density of approximately 20 μmol /m^2^/s emitted from cool fluorescent light tubes at 25 ± 1 °C. As described above, PCR and sequencing were used to identify the positive calli and positive buds.

### 2.4. Agrobacterium-Mediated Transient Transformed and GUS Histochemical Assay

The mature leaves over the fourth internode of the eucalyptus stem were selected as the materials. The lower epidermis was damaged by sandpaper friction and the leaf discs with a diameter of ten millimeters on both sides of the main leaf veins were taken with a punch, soaked in 0.2% Triton X-100 for 30 min, rinsed with sterile water, and then dried with sterile filter paper. The leaf discs were immersed in the *Agrobacterium* solution with an OD600 of 0.6~0.8, vacuumed at −0.05 MPa for 10 min, and infected for 30 min after they had completely sunk into the liquid. The leaf discs were taken out and placed on sterile filter paper saturated with a 1/2 MS liquid medium (containing 100 mg/L of vitamin C) and cultured in the dark at 28 °C for 48 h. The GUS histochemical assay was performed according to the method of Jefferson et al. [27].

### 2.5. Image-Based Expression Analysis of GUS Staining

Different treatment combinations of leaf discs were acquired with a Sony digital camera (Sony DSC-T20, Tokyo, Japan). A photographic device was used to fix the camera directly above a vertical eucalyptus leaf disc, positioned 30 cm away from the height of the leaf disc. The photographs were taken using the same camera settings (200-ISO, f/5.6, and 72 dpi).

### 2.6. Induction Treatment and qPCR Assays

Pathogen strains of *Phytophthora capsici* and *Ralstonia solanacearum* were purified and preserved in our laboratory. The TMV particles were prepared as described previously by Huang et al. [9]. After the infected leaf discs were cultured in the dark on sterile filter paper saturated with a 1/2 MS liquid medium for 24 h, spore suspensions were evenly spread on the surface of the leaf discs at 50 μL 1 × 10^6^ CFU (Colony-Forming Units) for *Ralstonia solanacearum* or *Phytophthora*, at 50 μL 2 mmol/L salicylic acid solution, or 50 μL extracts containing TMV particles. The leaf discs were cultured in the dark at 28 °C for 24 h, then ground into fine powder in liquid nitrogen, and the total RNA was extracted with Fruit Mate and an RNAiso Plus reagent (TakaraBio, Beijing, China). The total RNA quality was evaluated with a NanoDrop 2000C spectrophotometer (Thermo Fisher Scientific, Waltham, MA, USA). The total RNA was used as the template to complete reverse transcription to obtain the cDNA following the manufacturer’s instructions (PrimeScript RT reagent kit with gDNA eraser, Takara).

The transcriptional levels of the *gus* gene were evaluated by qPCR using a CFX 96 (Bio-Rad, Hercules, CA, USA) and SYBR Premix Ex TaqTM II (TakaraBio, Beijing) with the *eucons08* gene [28] as an internal reference. The qPCR primers were as follows: q*gus* F/R, 5′-CTGATAG CGCGTGACAAAAA-3′/ 5′-GGCACAGCACATCAAAGAGA-3′; eucons 08 F/R, 5′-TCCAATCCGAGTCGCTGTCATTGT-3′/5′-TGATGAGCCTCTCTGGTTTGACCT-3′. The thermal cycling conditions were as follows: initial denaturation step at 95°C for 30 s, 40 cycles of 9 s at 94 °C, 9 s at 55 °C, and 15 s at 72 °C. The specificity of the primer pairs was verified by melting curve analysis from 65 °C to 90 °C. The quantification of the relative changes in gene expression was performed using the 2^−ΔΔCt^ method as previously described by reference [29] and Bio-Rad CFX Manager software 3.1. The basal expression level of the original GWSF promoter (the CK in GWSF group) was normalized to 1. The relative expression level of four promoters under different induction treatments was calculated as the ratio between their actual levels and CK levels of GWSF.

### 2.7. Data Processing

Statistical analysis was carried out using SPSS 20.0 (IBM, Armonk, NY, USA) and a plot was produced using OriginPro (Version 2019b 9.6.5.169, Originlab, Northampton, MA, USA). All measurements were repeated three times.

## 3. Results

### 3.1. Results of GUS Histochemical Staining

**Figure 2:**
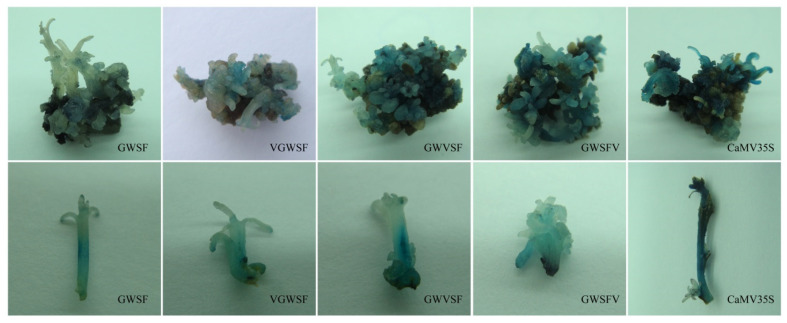
GUS histochemical staining for evaluation of the basal transcription activities of improved promoters in transgenic calli and adventitious buds of *E. urophylla*. GWSF was the original promoter. V-element dimers were inserted into the upstream (VGWSF), midstream (GWVSF), or downstream (GWSFV) of the original promoter (GWSF) to obtain three modified promoters, respectively. Results of transgenic calli (in the upper row) and adventitious buds (in the lower row) are showed.

The improved promoters were used to replace the *CaMV35S* promoter in the plasmid *pBI121*. The transcriptional activity of the improved promoters could be inferred by detecting the activity of glucuronidase by GUS histochemical staining or by quantitative assays [30]. The histochemical staining results of the adventitious buds indicated that the promoters had been successfully integrated into the *E. urophylla* genome and that they drove the expression of the *gus* gene to produce glucuronidase (Figure 2). Compared to *CaMV35S*, four artificial promoters displayed lower basal activities. There was a noticeable difference in the intensity of color between adventitious buds on the same callus block, as well as the intensity of color within the same adventitious bud (Figure 2). Clearly, there was an error in inferring the promoter activity from the color intensities of calli and adventitious buds.

**Figure 3:**
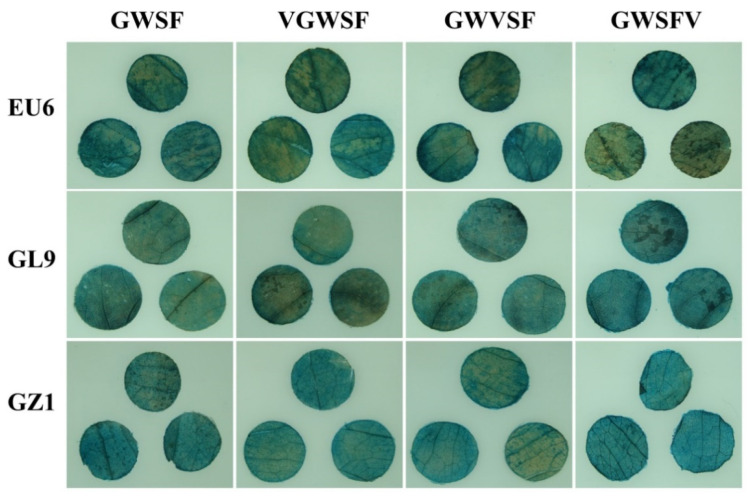
GUS histochemical staining evaluation of the basal transcription activities of the improved promoters in eucalyptus leaf discs. Leaf discs of eucalyptus were prepared as described in the materials and methods. EU6: *E. urophylla* U6; GL9: *E. urophylla* × *E. grandis* Guanglin 9; and GZ1: *E. urophylla* × *E. tereticornis* Guangzhou 1.

The leaf discs were cultured on filter paper saturated with a 1/2 MS liquid medium for 48 h by an *Agrobacterium*-mediated method. The recombined promoter drove the transcription of the *gus* gene, resulting in the accumulation of glucuronidase in the cells. As a result of the GUS histochemical staining, the four promoters showed a basal transcriptional level. In all three genotypes, including *E. urophylla* (EU6), *E. urophylla* × *E. grandis* (Guanglin 9), and *E. urophylla* × *E. tereticornis* (Guangzhou 1), the different promoters showed obvious differences in coloration (Figure 3). In all three kinds of eucalyptus leaf discs, the color intensity of the same promoter was different and the color intensities from high to low were Guangzhou 1, Guanglin 9, and EU6. There was no doubt that the insertion of the V-element altered the basal level of the promoter. A V-element was inserted into the downstream region of the original promoter to increase the basal level of GWSFV significantly. Moreover, when it was inserted into the upstream or midstream region of the original promoter, the chimeric promoter’s basal level was relatively low compared to the original promoter’s.

### 3.2. GUS Gene Expression Activity of Promoters Based on Color Feature Analysis

Using Sigmascan pro5.0 software (Systat Software Inc., USA), the color characteristic values of the leaf image were extracted to obtain the RGB values, namely red (R), green (G), and blue (B). As only the blue (B) value varies among the combinations, this study utilized an average B value per unit area in order to evaluate the GUS gene expression. Table 1 presents a two-factor model of the B values. Its *p*-value is 2.04 × 10^−6^, which is less than 0.001, indicating that the model is feasible and reliable. The B factor (genotype) has a much smaller *p*-value than the A factor (promoter), indicating a greater difference between the levels of the B factor than the A factor. Two factors significantly influenced the B values (the GUS gene expression activity), and their interaction also significantly affected the B values.

**Figure 4:**
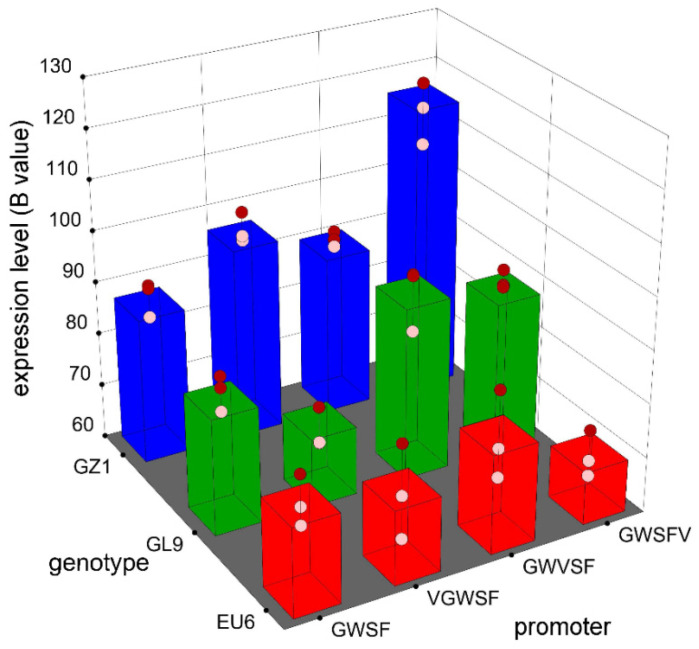
GUS gene expression in three eucalyptus genotypes from different promoters. Blue values of extracted RBG were measured by GUS histochemical staining from three eucalyptus leaves after three measurement replicates. According to the established two-factor full factorial model, B value (blue value) of the column in the figure reflects the predicted value from the model function. The red circles and light red circles in the figure are design points above the predicted value and below the predicted value, respectively. A factor consists of four promoters, namely GWSF, VGWSF, GWVSF, and GWSFV. B factor is a genotype factor, which is composed of three genotypes of GZ1, EU6, and GL9.

The established two-factor model shows the predicted B values of different treatment combinations in Figure 4. Generally, GZ1 had a larger B value than the other two genotypes. Among the four promoters, there is a relatively large difference. A comparison of the B values of the different combinations indicated that in EU6, only the GWVSF promoter was larger than the original promoter (GWSF); in GL9, only the VGWSF promoter was smaller than the original promoter (GWSF); in GZ1, three improved promoters were larger than the original promoter, and the GWSFV promoter had a maximum value.

As a result, we believe that the B values were influenced by the genotype and promoter type, and the combination of the GWSFV promoter in GZ1 represented the highest GUS gene expression activity in eucalyptus leaf discs.

### 3.3. Assays of Improved Promoters for Transcriptional Activities 

GUS (β-glucuronidase) is particularly popular as a reporter gene for assaying endogenous promoter activity in plant tissues via histochemical staining, but this can only approximate the transcriptional level of the promoter, whereas qPCR can directly quantify the relative transcriptional activities of the promoters.

**Figure 5:**
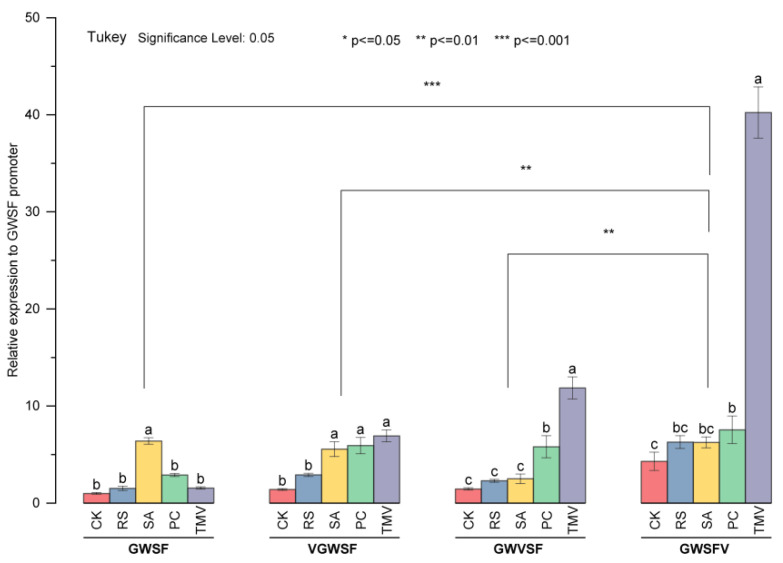
The relative expression levels of four promoters in *E. urophylla*. Leaf discs with transient expression were assayed for promoter activity by quantitative PCR. The basal transcriptional level of the original GWSF, namely the CK in the GWSF group, was normalized to 1. CK: basal transcription level of the corresponding promoter; SA: salicylic acid; RS: *Ralstonia solanacearum*; PC: *Phytophthora capsici*; and TMV: tobacco mosaic virus. The experiments were performed at least three times with similar results. The values of the bars are mean values. In the figure, different small letters indicate that different treatments in the same promoter group differ significantly at the level of 0.05. In the upper part of the figure, ** and *** indicate significant differences among the promoter groups at the level of 0.01, and 0.001, respectively. The statistics identified above are based on the Tukey test.

In this study, *E. urophylla*, with significantly differing GUS histochemical staining between different promoters, was used as the experimental material, and we further investigated the basal and inducible transcriptional levels of the three improved promoters by qPCR. In *E. urophylla* leaf discs, the basal level of the GWSF promoter, i.e., the CK in the GWSF group, had been normalized to 1 (Figure 5). The basal levels of VGWSF, GWVSF, and GWSFV assayed by qPCR were 1.40, 1.45, and 4.15, respectively (Figure 5), and the qPCR results were consistent with the staining results in Figure 3 and Figure 4.

Three improved promoters obtained by inserting the V-element all have the properties of being induced by salicylic acid, *Ralstonia solanacearum*, and *phytophthora*. The induced levels were between 2.30 and 7.66 folds (Figure 5). Obviously, the insertion of new elements will not cause the loss of the inducibility of the original promoter. The transcriptional activity of GWSF induced by TMV was 1.55, which was slightly higher than the basal level of the GWSF promoter, and the response to the TMV induction was not obvious. The transcriptional activities of VGWSF, GWVSF, and GWSFV induced by TMV were 6.94, 11.89, and 40.61, respectively. Improved promoters with V-elements demonstrated a significantly higher TMV induction activity. In comparing the three improved promoters, it was found that the induction activity from high to low was GWSFV > GWVSF > VGWSF, and their activities were significantly varied, indicating that the closer the V-element was to the *CaMV35S* minimal promoter, the greater the inducing activity.

## 4. Discussion

In transgenic disease-resistant breeding, the continuous expression of resistance genes will lead to developmental delays and reduced yields [31]. Choosing an appropriate promoter to precisely regulate the expression of resistance genes can eliminate such side effects. However, the transcriptional properties of natural promoters are limited [32]. In recent years, many results have been achieved in the research on artificial promoters [33,34]. However, it is less well understood how the basal levels and inducible activities of artificial promoters are affected by new elements [35]

The GWSF promoter element has the advantage of a low basal expression, and it can be induced by *Ralstonia solanacearum*, *Phytophthora*, salicylic acid, etc. [7]. In this study, V-element dimers with a virus-inducing activity were inserted into a different region of the GWSF sequence to evaluate the impact of introducing V-elements on the original promoter’s transcriptional capabilities, and the positional effect of inserting V-elements on the transcriptional properties of the original promoter. Meanwhile, the research investigated whether V-elements might provide a better virus-inducible activity of the promoter.

Three improved promoters, namely VGWSF, GWVSF, and GWSFV, were designed by inserting a V-element dimer into the upstream, middle, and downstream regions of the GWSF promoter sequence, respectively. Both the GUS histochemical staining and qPCR results implied that the insertion element had an obvious position effect on the transcriptional properties of the original promoter. The closer the cis-element is to the *CaMV35S* minimal promoter, the greater the transcriptional activity. The above results confirmed Rushton’s viewpoint that spacing between elements might be crucial for promoter activity, and transcriptional activity was enhanced when the cis-element was located near the minimal/core promoter [14]. 

The basal level of three promoters increased significantly when a V-element was inserted into the downstream region of the GWSF sequence. However, the basal level did not change significantly when a V-element was inserted into the upstream or midstream region. The insertion of new elements will not cause the loss of the original inducible properties of the promoters. The three promoters obtained by inserting the V-element all had the properties of being induced by salicylic acid, *Ralstonia solanacearum*, and *Phytophthora*, and the induced level was between 2.30 and 7.66 (Figure 5). The TMV induction increased the transcriptional activity of GWSF by 1.5 times compared to its basal level. The transcriptional activities of VGWSF, GWVSF, and GWSFV when induced by TMV were 6.94, 11.89, and 40.61, respectively. Improved promoters with the V-element were significantly more TMV-inducible, and the closer the V-element was to the *CaMV35S* minimal promoter, the greater the virus-inducible activity. However, it remains to be determined whether the activity trend exists in improving other promoters as well as how close the cis-element is to the minimum/core promoter.

Eucalyptus species are recalcitrant to in vitro organogenesis, and even more to genetic transformation, so much effort was required to obtain a few events [3]. As eucalyptus explants are prone to browning during the tissue culture process, it is more challenging to implement eucalyptus transgenes that were derived from a tissue culture. We found in previous studies that 2-Cl-PBU inhibited callus browning and promoted adventitious bud differentiation in *E. urophylla* by repressing *rboh1* transcription [36]. The SPCa medium supplemented with 3.99 μmol/L of PBU, 0.57 μmol/L of BAP, and 0.57 μmol/L of IAA was highly efficient for callus induction and adventitious bud differentiation. In transgenic eucalyptus plants screened with kanamycin, GUS staining indicated that the promoters integrated successfully and drove the *gus* expression. However, adventitious buds on the same callus block showed different colors for GUS staining, as did parts of the same adventitious bud. Our hypothesis was that the reason might be the formation of chimeras, which was the biggest challenge encountered during the development of transgenic woody plants. Undoubtedly, it is difficult to infer the promoter activity from the color intensity on the GUS staining of callus and adventitious buds. This study optimized the *Agrobacterium*-mediated method to estimate the promoter transcription activity in eucalyptus leaf discs. Sandpaper friction damaged the epidermis, Triton X-100 soaking reduced the lipid barrier, vacuuming promoted penetration, vitamin C reduced browning; and co-cultivation in the dark was conducive to gene expression. Meanwhile, the image digitization method was applied to extract the color features of the leaf discs, and the key color feature value responding to the treated combination was selected for modeling analysis. As a result of the application and optimization of the above-mentioned test methods, it is possible to quickly and accurately determine the transcriptional activity of a promoter in eucalyptus leaves.

The basal level of GWVSF obtained in this experiment was 1.45 times the basal level of the original GWSF promoter. Furthermore, the transcriptional activity of the GWVSF induced by salicylic acid, bacteria, fungi, and viruses was 2.49, 2.30, 5.86, and 11.89, respectively. It not only retained the advantages of low basal levels and being induced by salicylic acid, bacteria, and fungi, but also obtained TMV inducibility, which could be used for plant transgenic disease-resistant breeding. Using GWVSF to drive the expression of broad-spectrum disease resistance genes was expected to obtain transgenic varieties with broad-spectrum disease resistance. Our study optimized the transient expression and genetic transformation of eucalyptus. This work might be useful in designing and optimizing artificial promoters.

## 5. Conclusions

The expression activities of three improved promoters by inserting a V-element dimer into the different regions of the GWSF promoter sequence were detected by GUS staining. Furthermore, color intensity differed significantly within and between adventitious buds.

The GUS gene expression activity (B values) was significantly affected by the genotype and promoter factors by color feature extraction and analysis of GUS staining. Using color feature extraction and the analysis of leaf discs, genotype and promoter factors were significantly associated with GUS gene expression activity.

The different promoters of transgenic plants were treated by plant pathogenic induction, and the results of qPCR assays showed that: the basal level of the improved promoters increased significantly; the improved promoters had inducible responses to more inducers; the insertion element had an obvious position effect on the transcriptional properties of the original promoter; and the closer the V-element was to the *CaMV35S* minimal promoter, the higher the virus-inducible activity.

## Figures and Tables

**Table 1:** Modeling statistical analysis of the mean blue value of RGB extracted from transgenic eucalyptus leaves by GUS histochemical staining. In the table, sources: variation sources; df: degree of freedom; and *p*-value: the probability of the same line item. If the *p*-value is less than 0.001, it indicates a significant difference at a level of 0.001. Block: different treatment groups; model: two-factor full factorial model; A×B: interaction of A factor (promoter) and B factor (genotype); and residual: residual error of the model.

Sources	Sum of Squares	df	Mean Square	F-Value	*p*-Value
Block	919.42	8	114.93		
Model	4720.38	11	429.13	15.06	2.04 × 10^−6^
A factor: promoter	482.50	3	160.83	5.64	7.81 × 10^−3^
B factor: genotype	2889.12	2	1444.56	50.68	1.19 × 10^−7^
A × B	1348.77	6	224.79	7.89	4.50 × 10^−4^
Residual	456.04	16	28.50		

## Data Availability

Not applicable.
